# Supplementary material for: Dendrimer Nanodevices and Gallic Acid as Novel Strategies to Fight Chemoresistance in Neuroblastoma Cells
Source: Nanomaterials (Basel). 2020 Jun 26;10(6):1243. doi: 10.3390/nano10061243 (PMC7353457; doi:10.3390/nano10061243)
Supplement: Supplementary file 1 [file nanomaterials-10-01243-s001.pdf]

nanomaterials

Supplementary Materials

Dendrimer Nanodevices and Gallic Acid as Novel Strategies to Fight Chemoresistance in Neuroblastoma Cells

S. Alfei [1,*], B. Marenco [2], G. Zuccari [1], F. Turrini [1], and C. Domenicotti [2]

[1] Department of Pharmacy, University of Genoa, Viale Cembrano, 4 I-16148 Genoa, Italy

[2] Department of Experimental Medicine - DIMES, Via Alberti L.B. 2 I- 16132 Genoa, Italy

*Corresponding Author: Prof. Silvana Alfei
Department of Pharmacy, University of Genoa
Phone number: +39-010-3532296
Fax number: +39-010-3532684
Email: alfei@difar.unige.it
ORCID: 0000-0002-4630-4371

Table of Contents

Figure S1. Structure of dendron intermediates prepared to synthetize **4**: D4BnA, D4BnOH, D5BnA and D5ACOOH.

Figure S2. Morphology, size and Z-potential of GAD by SEM and DLS analysis.

Section S1. Characterization data of dendrimer 4 and GAD 6

FTIR, NMR spectra data and Elemental analysis results of compound **4**.

FTIR, NMR spectra data and Elemental analysis results of GAD **6**.

Copies of FTIR and NMR spectra of dendrimer 4 and GAD 6

Figure S3. FTIR spectrum (KBr) of dendrimer **4**.

Figure S4. ^{1}H NMR spectrum (DMSO-*d6*, 300 MHz) of dendrimer **4**.

Figure S5. ^{13}C NMR and DEPT-135 spectra (DMSO-*d6*, 75.5 MHz) of dendrimer **4**.

Figure S6. FTIR spectrum (KBr) of GAD **6**.

Figure S7. ^{1}H NMR spectrum (DMSO-d_6, 300 MHz) of GAD **6**.

Figure S8. ^{13}C NMR and DEPT-135 spectra (DMSO-d_6, 75.5 MHz) of GAD **6.**

Table S1. Molecular Weight (MW) and significant physicochemical data of dendrimer **4** and GAD **6**.

Scheme SI. Synthesis of the protected/activate GA-derivative GA-TBDMS-Cl.

Section S2. Antioxidant activity of GAD 6

Figure S9. RSA (%) curves recorded at different concentrations of dendrimer GAD **6**, GA, AA and Trolox in ethanol or water solution, expressed in mM.

Figure S10. Comparison between radical scavenging activity expressed as IC_{50} (mM) of GAD, GA, Vitamins C and E and Trolox.

Figure S11. GAD inhibition of peroxide formation in samples of *β*-pinene (a) and *Pinus Mugo* essential oil (b) subjected to thermal induced oxidative degradation.

Figure S12. Intra-platelets ROS production inhibition activity of GAD and GA expressed as IC_{50} (μM).

Section S3. FTIR and NMR spectra of gallic acid 1

Figure S13. FTIR spectrum (KBr) of **1**.

Figure S14. ^{1}H NMR spectrum ($CDCl_3$/DMSO-*d6*, 300 MHz) of **1** [CAS Registry Number: 149-91-7 - Source: Sigma-Aldrich (Spectral data were obtained from Advanced Chemistry Development, Inc.)].

Figure S15. ^{13}H NMR spectrum ($CDCl_3$/DMSO-*d6*, 75.5 MHz) of **1** [CAS Registry Number: 149-91-7 - Source: Sigma-Aldrich (Spectral data were obtained from Advanced Chemistry Development, Inc.)].

Section S4. Qualitative investigations on GALD 7: $FeCl_3$ test result, FTIR and NMR

Figure S16. (**a**) Pale yellow ethanol solution of GALD before $FeCl_3$ test; (**b**) dark blue coloration of solution after the addition of $FeCl_3$ solution.

Figure S17. FTIR spectrum (KBr) of GALD **7**.

Figure S18. ^{1}H NMR spectrum (DMSO-*d6*, 300 MHz) of GALD **7**.

Section S5. Comparison between FTIR and ^{1}H NMR spectra of GA, dendrimer 4 and GALD 7

Figure S19. FTIR spectra of GA (green), dendrimer **4** (red) and GALD complex **7** (black) with in evidence the significant peaks.

Figure S20. ^{1}H NMR spectra (DMSO-*d6*) of (**a**) GA (300 MHz), (**b**) dendrimer **4** (300MHz) and (**c**) GALD **7** (300 MHz).

Section S6. Principal Components Analysis Results

Figure S21. Bi-plot on Components PC1 and PC2 (**a**); extrapolation of vectors on PC2 to estimate GA loading (%) (**b**).

Figure S22. Bi-plot on Components PC1 and PC2 including spectral data of non-complexed molecules isolated as solid from MeOH.

Section S7. UV-Vis determination of GA concentration in GALD

Table S2. Values of A, C_{GA} and ε_{GAOxC} obtained for the six aliquots of a 31.8 μg/mL sample of GALD **7**.

Table S3. Data of the calibration curve: $A_{average}$ and GA standards concentrations (C_{GA}), GA predicted concentrations (C_{GAp}), residuals, absolute percentage errors and C_{GA} (μM).

Figure S23. Standard GA calibration curve.

Figure S24. Real GA concentrations (C_{GA}) *versus* predicted ones (C_{GAp}).

Figure S25. Absorbance (A) at λ = 760 nm *versus* standards GA concentrations (μM).

Table S4. Statistical predictive concerning calibration set, significant data of calibration, errors in the calibration and correlation coefficients.

Equations S1, S2 and S3

Section S8. Dynamic Light Scattering Analysis Results

Figure S26. Dynamic Light Scattering Analysis of GALD **7**: multimolecular aggregates (megamers).

Figure S27. Dynamic Light Scattering Analysis of GALD **7**: unimolecular dendrimer particles and multimolecular aggregates (megamers).

Figure S28. Dynamic Light Scattering Analysis of GALD **7**: Z-potential.

References

D4BnA

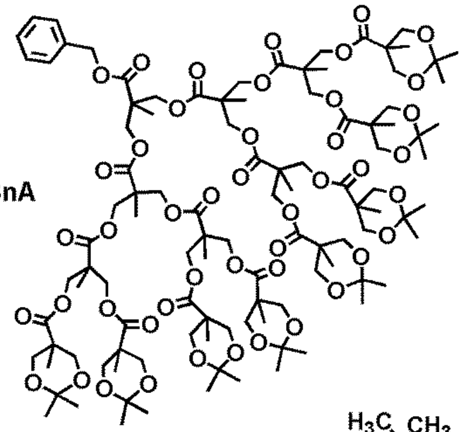

D4BnOH

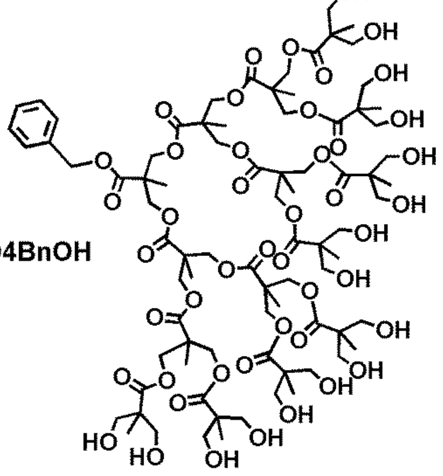

D5BnA

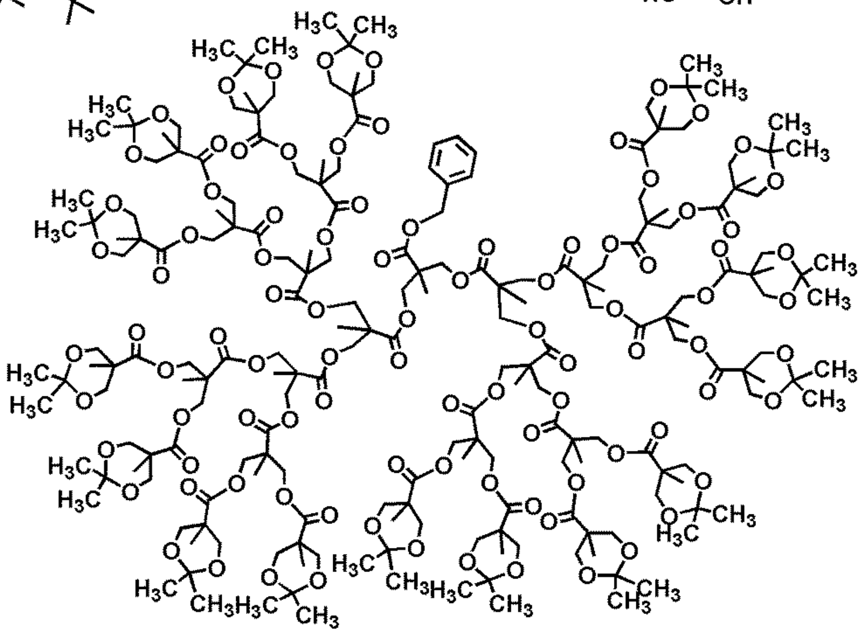

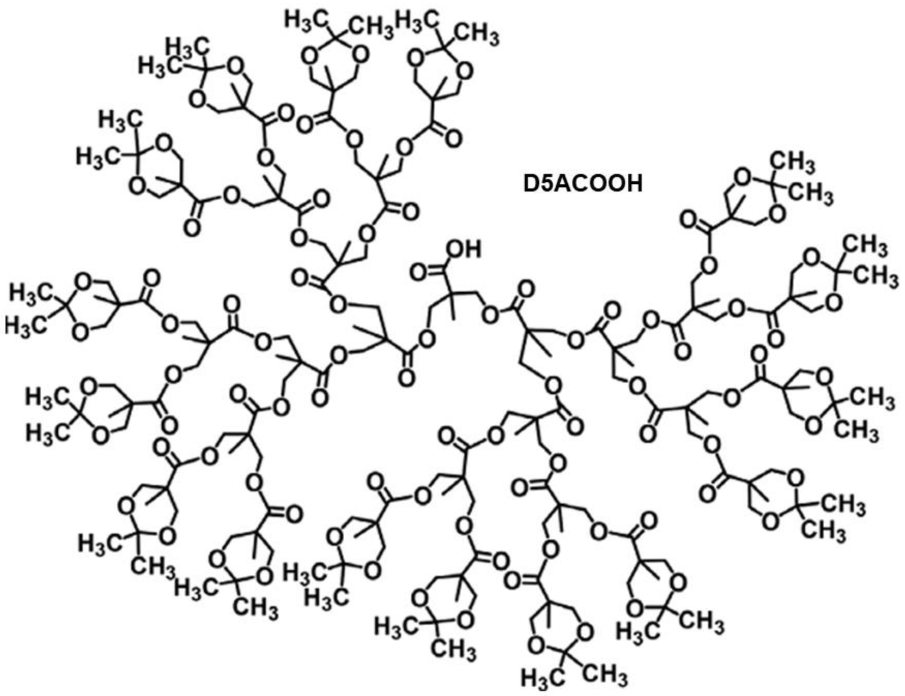

Figure S1. Structure of dendron intermediates prepared to synthetize **4**: D4BnA, D4BnOH, D5BnA and D5ACOOH [1,2].

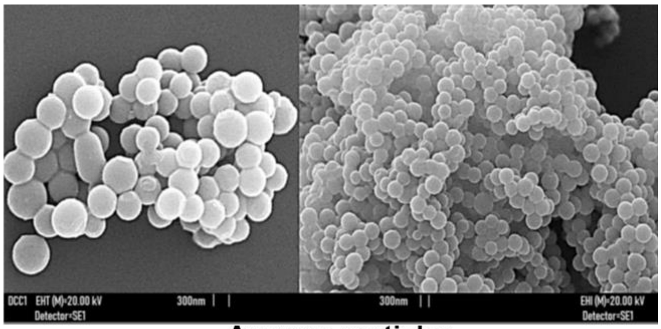

Z-AVE size (nm)^{a,b}	Average particles size (nm) by SEM analysis	Z-potential (mV)^{a,b}
375±7.9	387±11	-25±0.34

^{a}N = 12; ^{b}by DLS analysis

Figure S2. Morphology, size and Z-potential of GAD by SEM and DLS analysis [3-5].

Section S1. Characterization data of dendrimer 4 and GAD 6

FTIR, NMR spectra data and Elemental analysis results of compounds **4** [3]

*Dendrimer **4***. FTIR (KBr, cm^{-1}): 3436 (OH), 2936, 1737 (C=OO). ^{1}H NMR (300 MHz, DMSO-d_6), δ (ppm): 1.01, 1.16, 1.18, 1.23, 1.34 (five s signals, 186H, CH_3 of generations), 1.70 (m, 2H, CH_2 propandiol), 3.52 (dd, 128H, CH_2OH), 3.56 (partially overlapped signal, 2H, CH_2O propandiol), 3.98 (partially overlapped signal, 2H, CH_2O propandiol), 4.08-4.18 (m, 120H, CH_2O of four generations), 4.37 (br s, 64H, OH). ^{13}C NMR (75.5 MHz, DMSO-d_6) δ (ppm): 173.94, 171.73 (C=O), 64.27, 63.55 (CH_2O), 50.13 (quaternary C of fifth generation), 46.12 (other generation

detectable quaternary C), 17.05, 16.61 (CH_3 of generations). Found: C, 51.71; H, 7.01. $C_{313}H_{504}O_{188}$ requires C, 51.67; H, 6.98%.

FTIR, NMR spectra data and Elemental analysis results of GAD **6** [3]

GA-loaded dendrimer **6.** FTIR (KBr, cm^{-1}): 2932, 2899, 2861 (CH_3 and CH_2 dendrimer matrix), 1741 (C=OO inner matrix), 1726 (peripheral conjugated C=OOGA). ^{1}H NMR (300 MHz, DMSO-d_6), δ (ppm): 1.01, 1.16, 1.18, 1.23, 1.34 (five s signals, 186H, CH_3 of generations), 1.70 (m, 2H, CH_2 propandiol), 3.95 (m, 128H, GA esterified CH_2O), 4.05-4.40 (m, 120H, CH_2O of four generations), 7.32 (s, 128H, GA phenyl CH=), 8.00-10.00 (br s, GA phenols OH). ^{13}C NMR (75.5 MHz, DMSO-d_6) δ (ppm): 173.94, 171.73 (C=O of dendrimer scaffold), 167.11 (C=O of GA), 148.80, 145.94, 124.67 (quaternary C of phenyl), 117.41 (CH= of phenyl), 64.27, 63.55 (CH_2O), 50.13 (quaternary C of fifth generation), 46.12 (other generation detectable quaternary C), 17.05, 16.61 (CH3 of generations). Found: C, 54.03; H, 4.89. $C_{761}H_{760}O_{444}$ requires C, 53.72; H, 4.51%.

Copies of FTIR and NMR spectra of dendrimer 4 (G5-PD-OH in the spectrum) and GAD 6 [3]

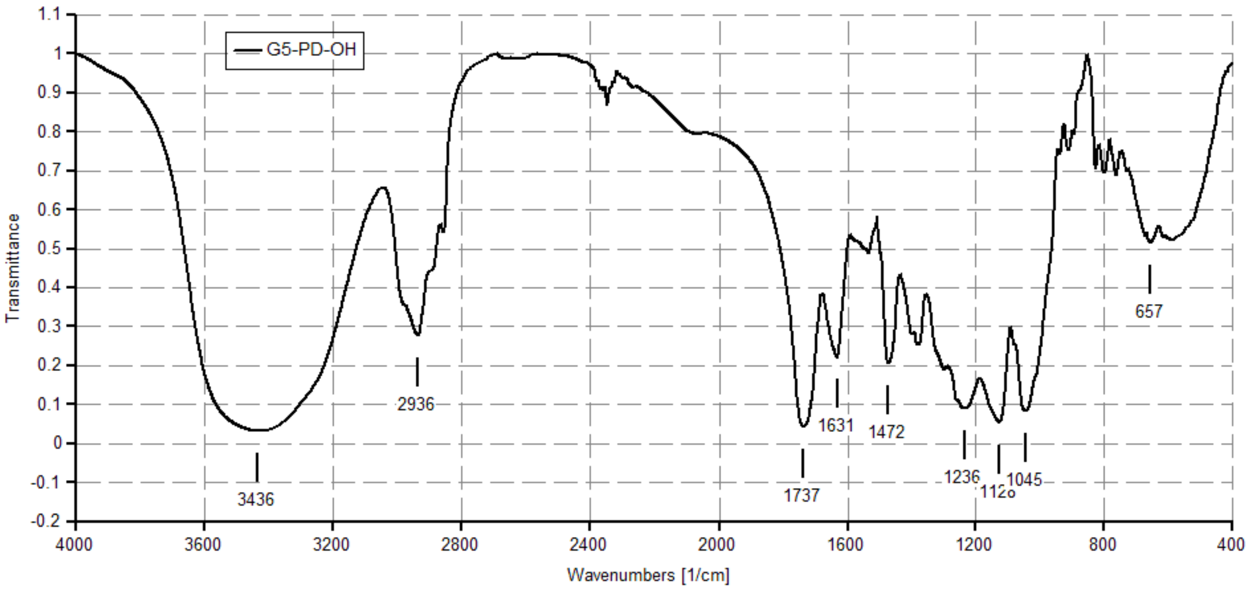

Figure S3. FTIR spectrum (KBr) of dendrimer **4.**

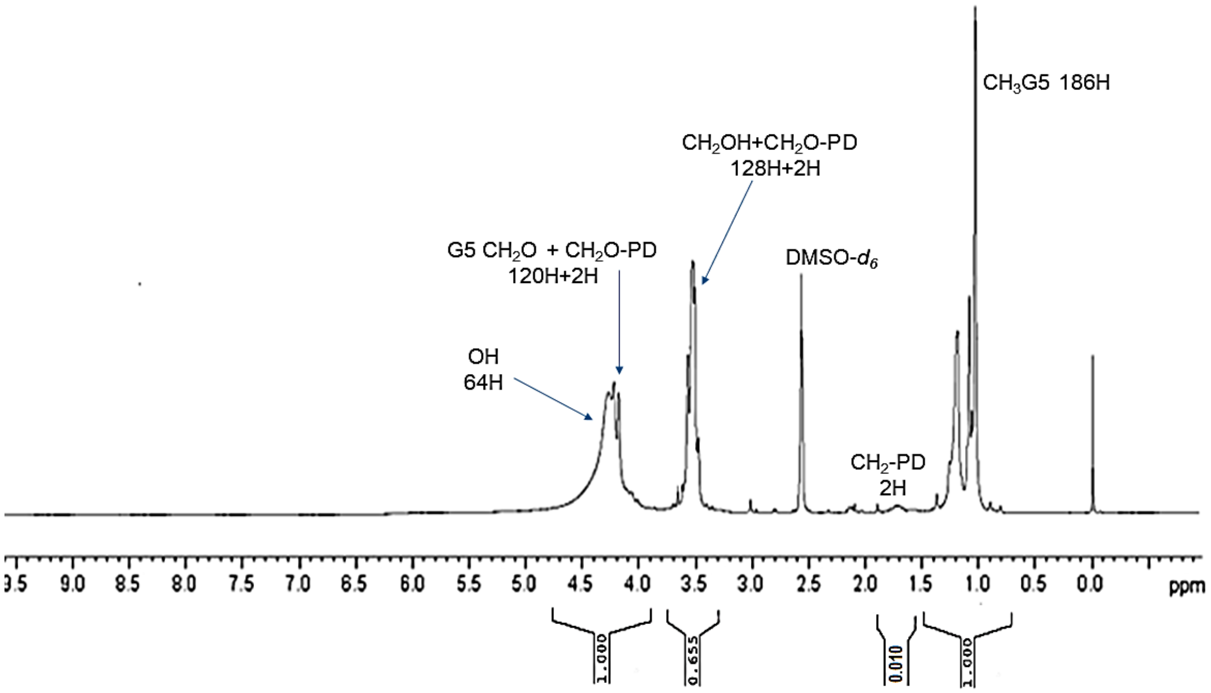

Figure S4. ^{1}H NMR spectrum (DMSO-*d6*, 300 MHz) of dendrimer **4.**

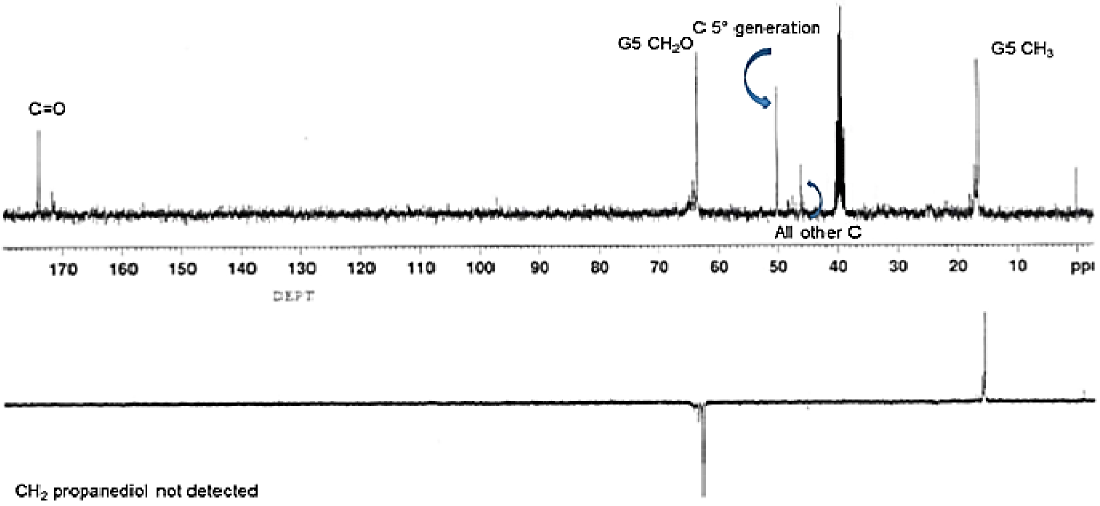

Figure S5. ^{13}C NMR and DEPT-135 spectra (DMSO-*d6*, 75.5 MHz) of dendrimer **4.**

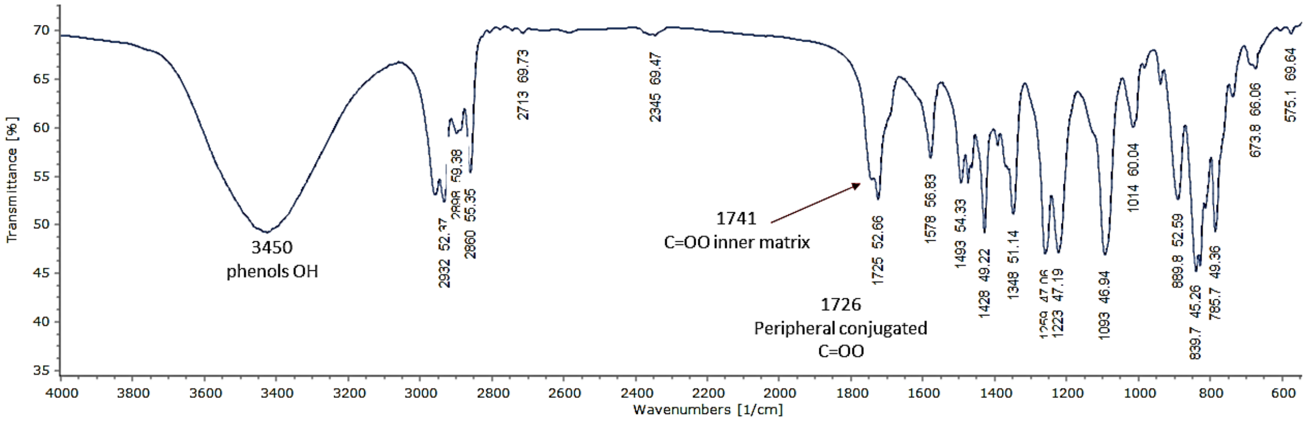

Figure S6. FTIR spectrum (KBr) of GAD **6**.

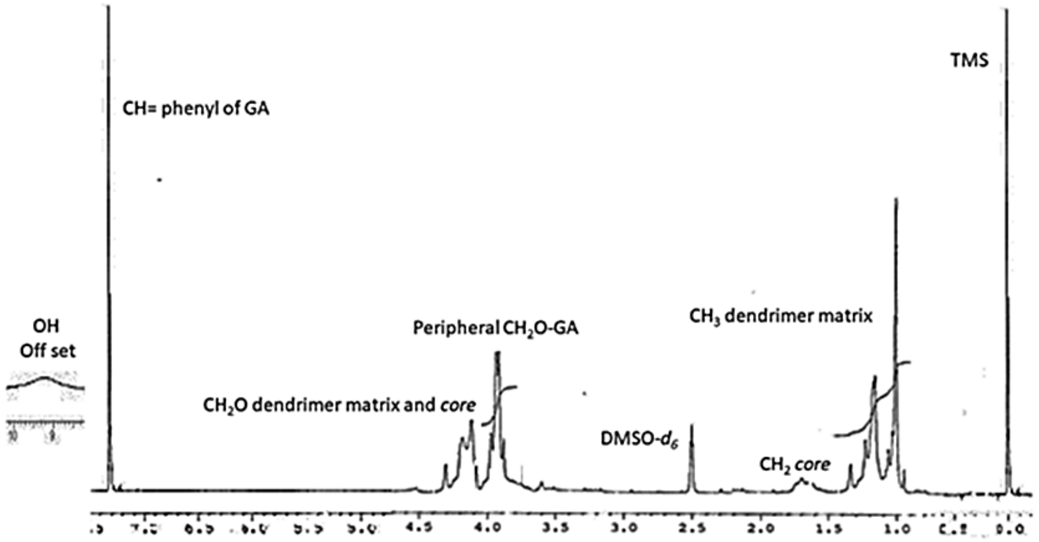

Figure S7. ^{1}H NMR spectrum (DMSO-d_6, 300 MHz) of GAD **6**.

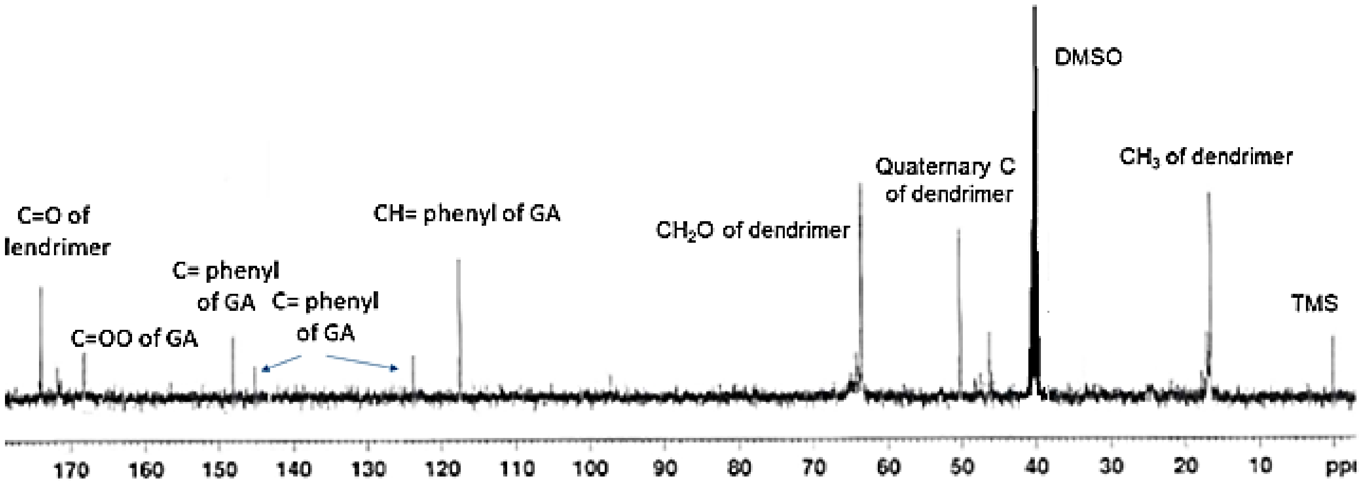

Figure S8. ^{13}C NMR and DEPT-135 spectra (DMSO-d_6, 75.5 MHz) of GAD **6**.

Table S1. Molecular Weight (MW) and significant physicochemical data of dendrimer **4** and GAD **6** [3].

Compound	Formula	MW	Required (%)	Found (%)	Error (%)	Physical state
**4**	$C_{313}H_{504}O_{188}$ [1]	7275.24 [1]	C 51.67<br>H 6.98	C 51.71<br>H 7.01	C 0.04<br>H 0.03	Fluffy white hygroscopic solid
**6**	$C_{761}H_{760}O_{444}$ [1]	17010.02 [1]	C 53.72<br>H 4.51	C 54.03<br>H 4.89	C 0.31<br>H 0.38	Brownish glassy hygroscopic solid

[1] Formulas and MW of dendrimer **4** and GAD **6** were estimated by ^{1}H NMR spectra and confirmed by Elemental Analysis.

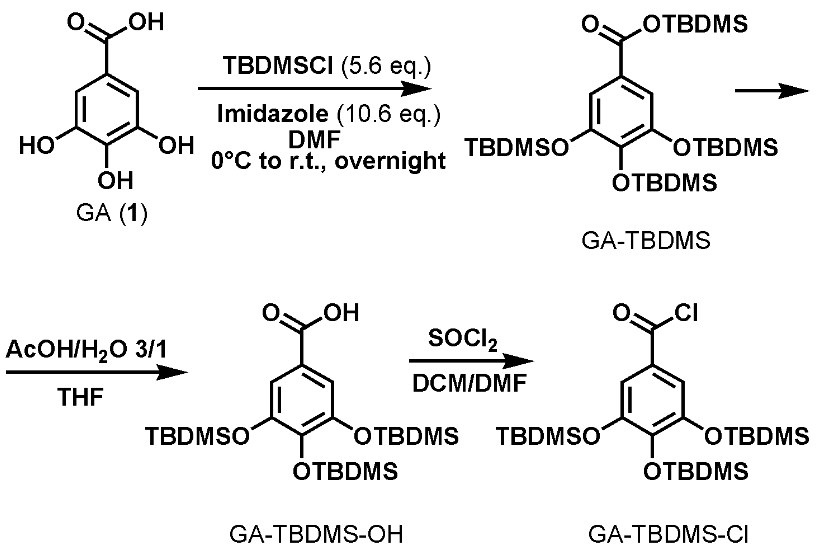

Scheme SI. Synthesis of the protected/activate GA-derivative GA-TBDMS-Cl.

Section S2. Antioxidant activity of GAD 6 [3-5]

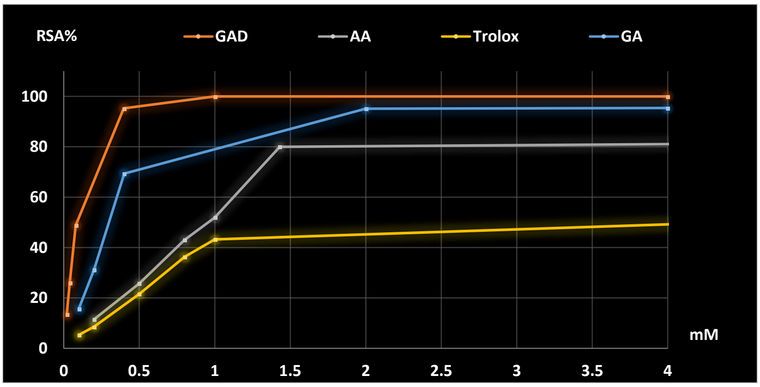

Figure S9. RSA (%) curves recorded at different concentrations of dendrimer GAD **6**, GA, AA and Trolox in ethanol or water solution, expressed in mM.

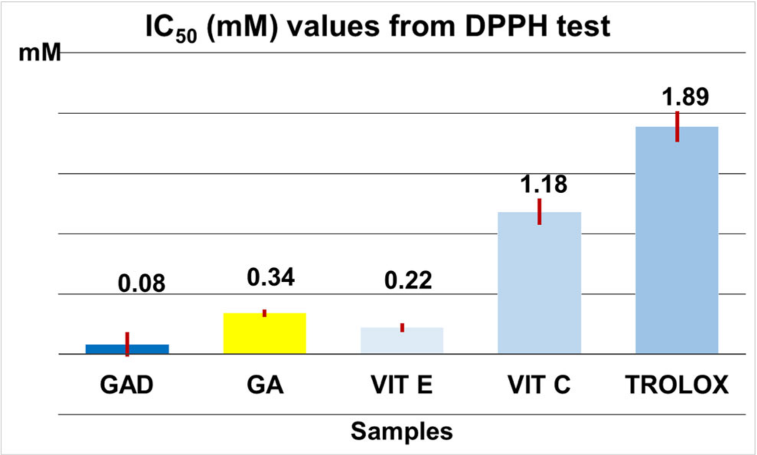

Figure S10. Comparison between radical scavenging activity expressed as IC_{50} (mM) of GAD, GA, Vitamins C and E and Trolox [3].

a) b)

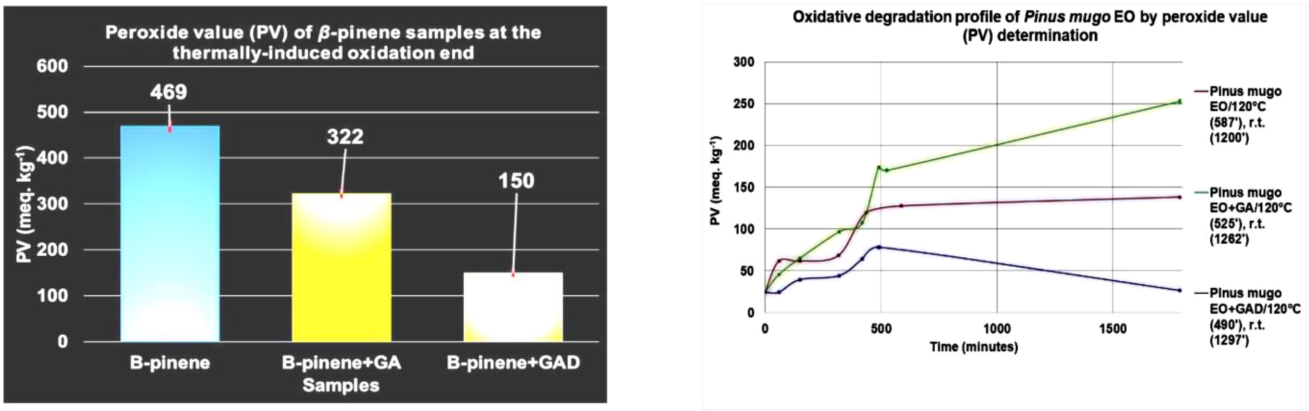

Figure S11. GAD inhibition of peroxide formation in samples of *β*-pinene (a) and *Pinus Mugo* essential oil (b) subjected to thermal induced oxidative degradation [4].

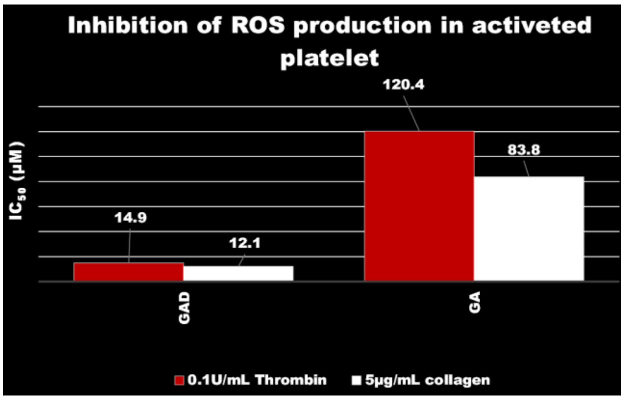

Figure S12. Intra-platelets ROS production inhibition activity of GAD and GA expressed as IC_{50} (μM) [5].

Section S3. **FTIR and NMR spectra of gallic acid (1)**

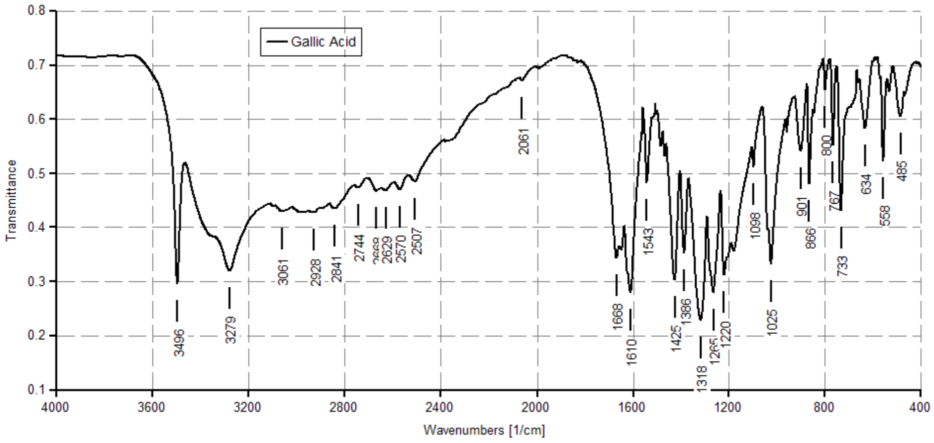

Figure S13. FTIR spectrum (KBr) of **1**.

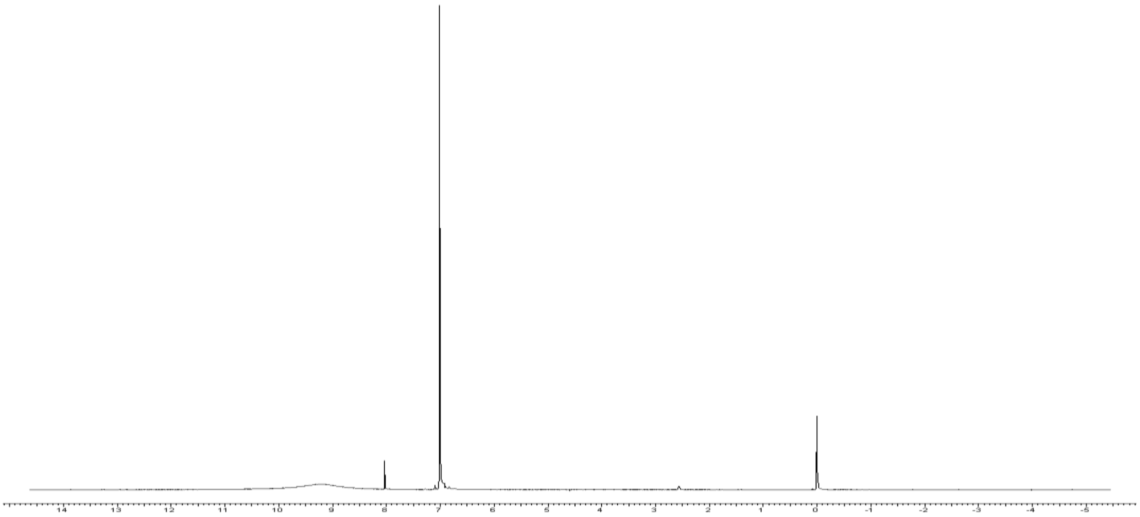

Figure S14. ^{1}H NMR spectrum ($CDCl_3$/DMSO-*d6*, 300 MHz) of **1** [CAS Registry Number: 149-91-7 - Source: Sigma-Aldrich (Spectral data were obtained from Advanced Chemistry Development, Inc.)].

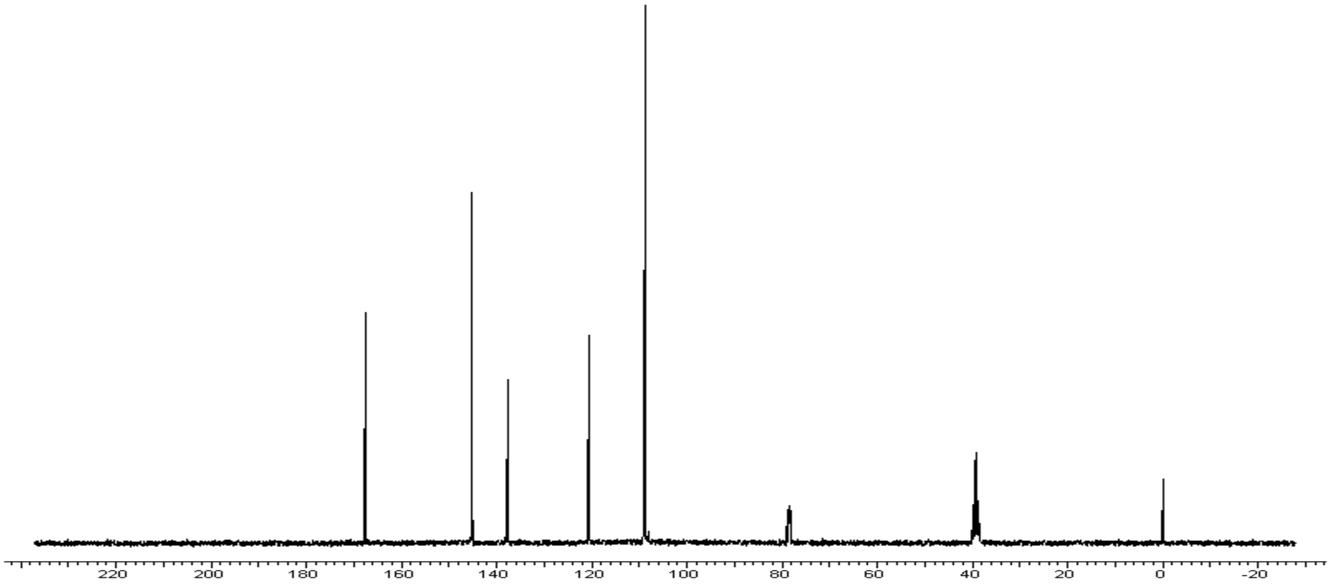

Figure S15. [13]H NMR spectrum ($CDCl_3$/DMSO-*d6*, 75.5 MHz) of **1** [CAS Registry Number: 149-91-7 - Source: Sigma-Aldrich (Spectral data were obtained from Advanced Chemistry Development, Inc.)].

Section S4. Qualitative investigations on GALD 7: $FeCl_3$ test, FTIR and NMR.

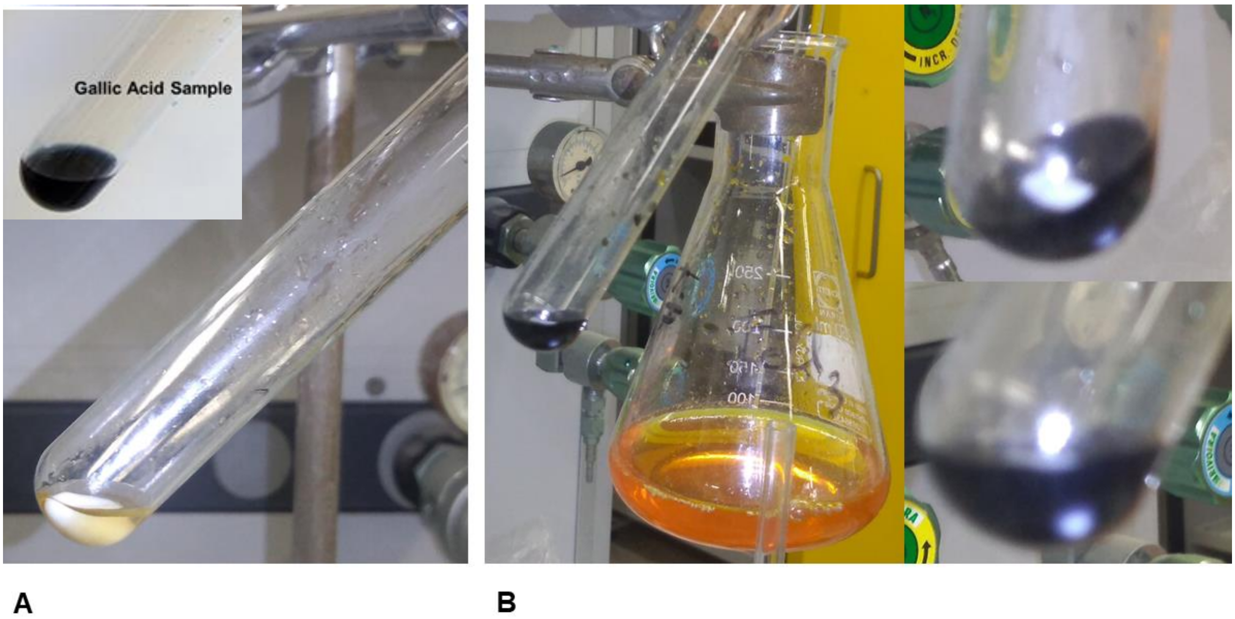

Figure S16. (**a**) Pale yellow ethanol solution of GALD before $FeCl_3$ test; (**b**) dark blue coloration of solution after the addition of $FeCl_3$ solution.

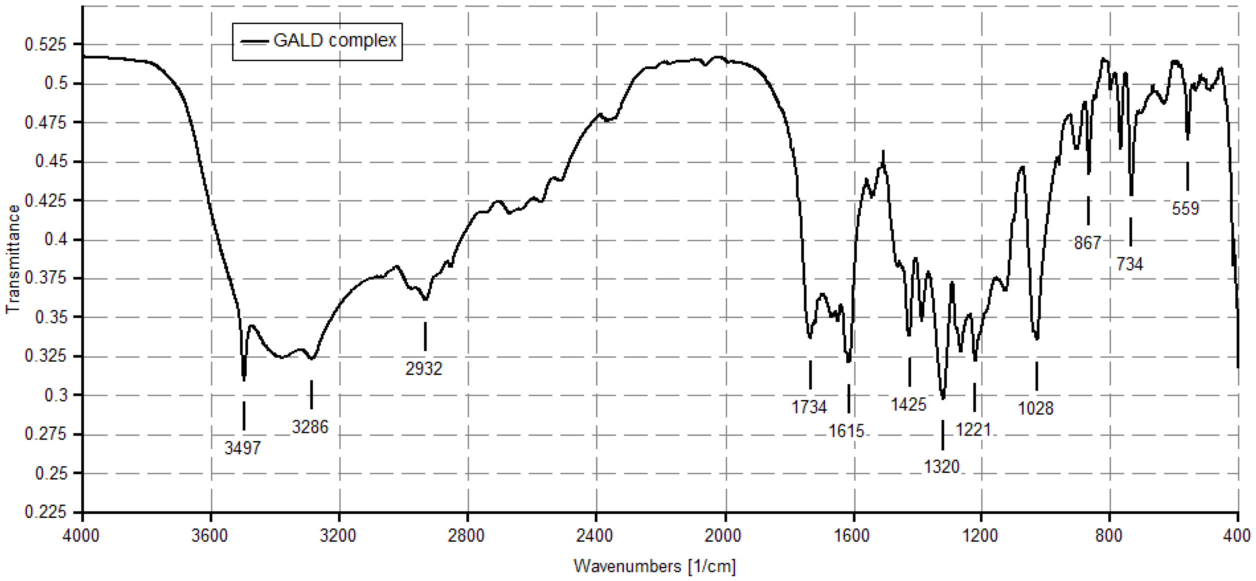

Figure S17. FTIR spectrum (KBr) of GALD **7**.

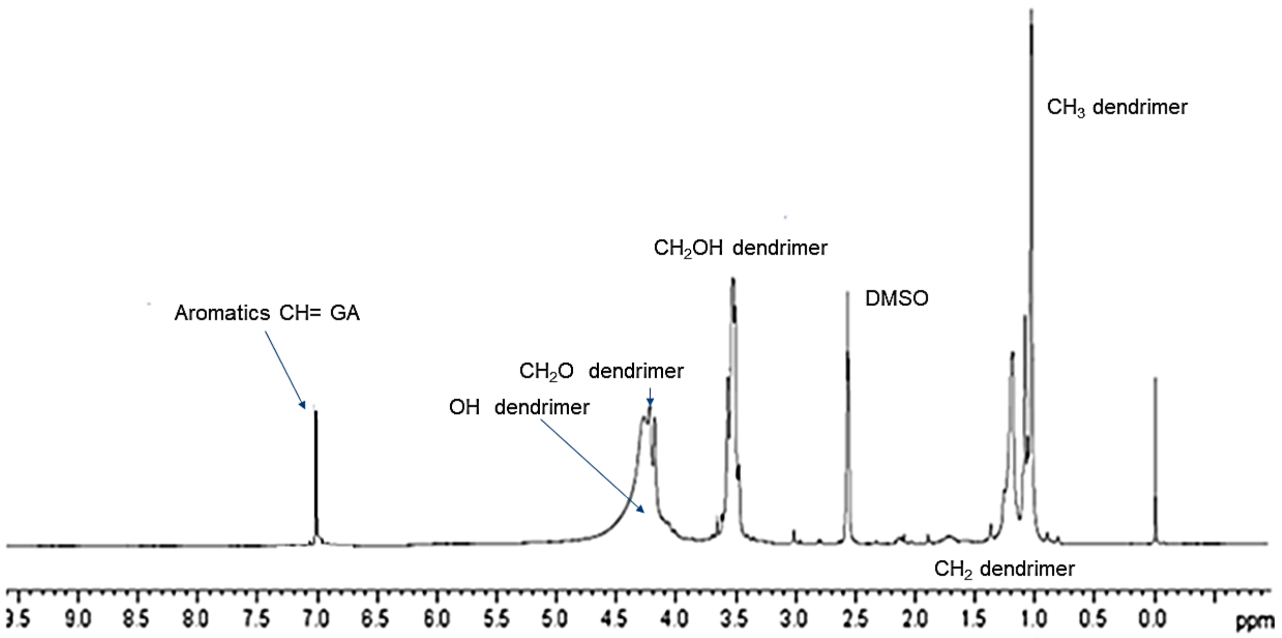

Figure S18. ^{1}H NMR spectrum (DMSO-*d6*, 300 MHz) of GALD **7**.

Section S5. Comparison between FTIR and NMR spectra of GA, 4 and GALD 7

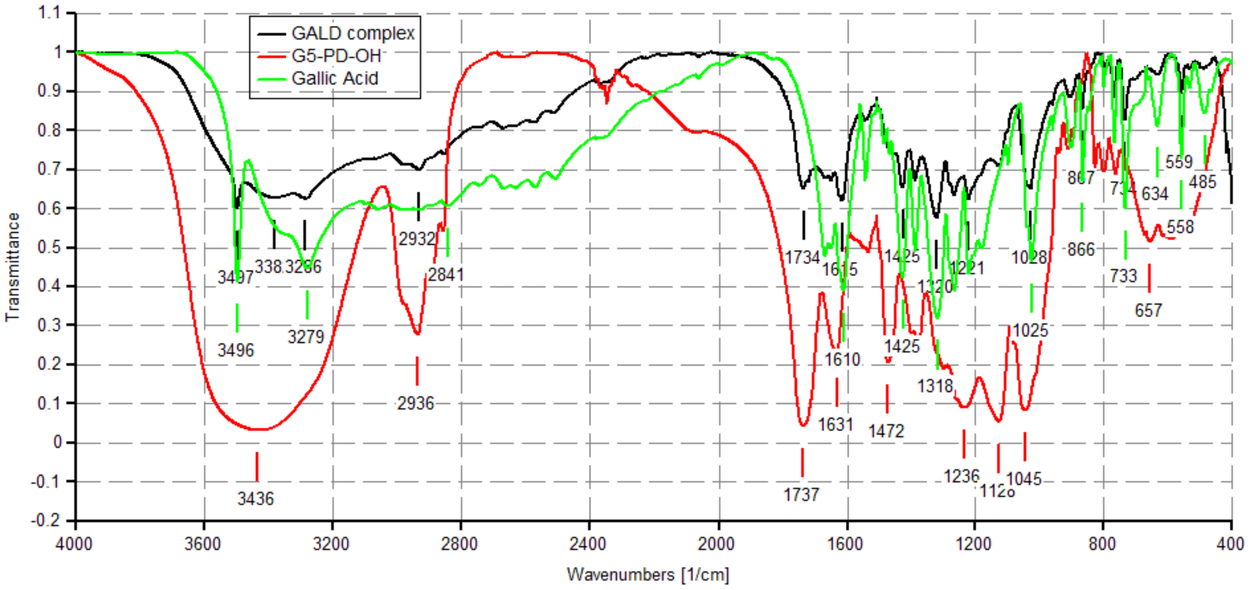

Figure S19. FTIR spectra of GA (green), dendrimer **4** (red) and GALD complex **7** (black) with in evidence the significant peaks.

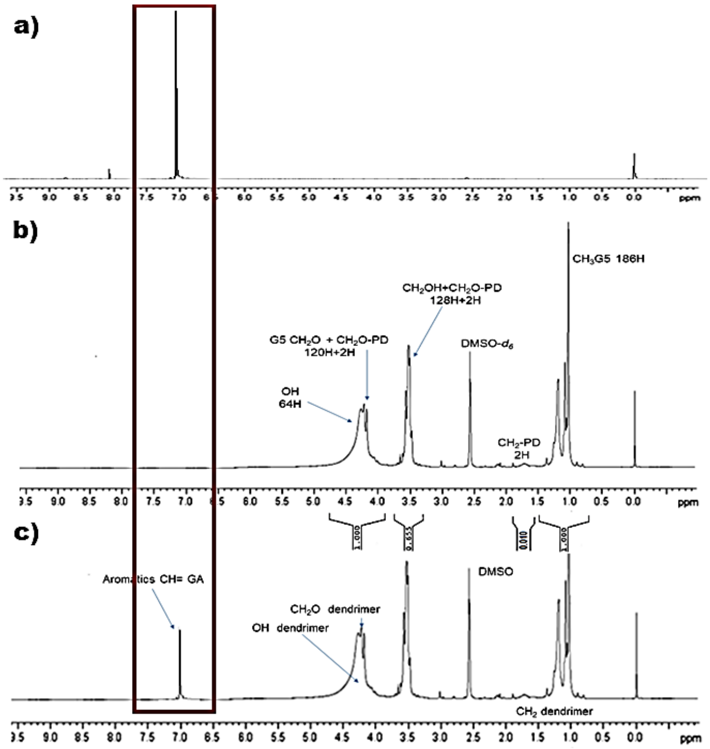

Figure S20. ^{1}H NMR spectra (DMSO-*d6*) of (**a**) GA (300 MHz), (**b**) dendrimer **4** (300MHz) and (**c**) GALD **7** (300 MHz).

Section S6. Principal Component Analysis results

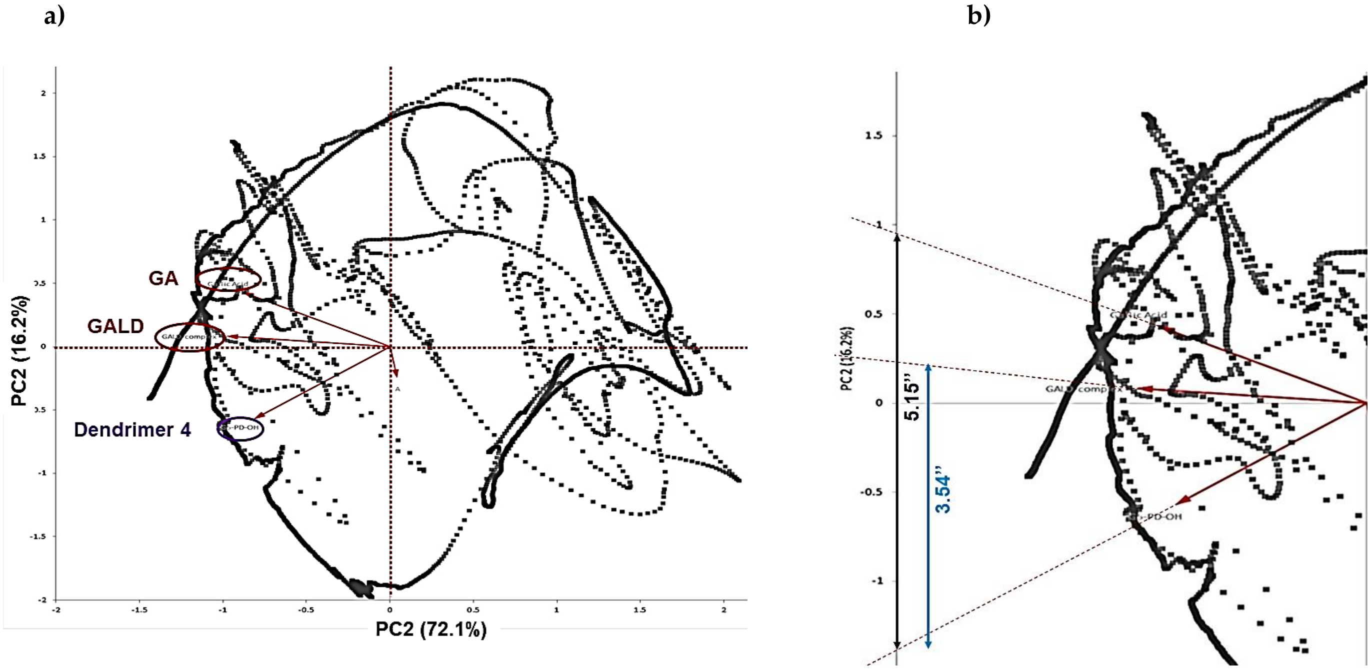

Figure S21. Bi-plot on Components PC1 and PC2 (**a**); extrapolation of vectors on PC2 to estimate GA loading (%) (**b**).

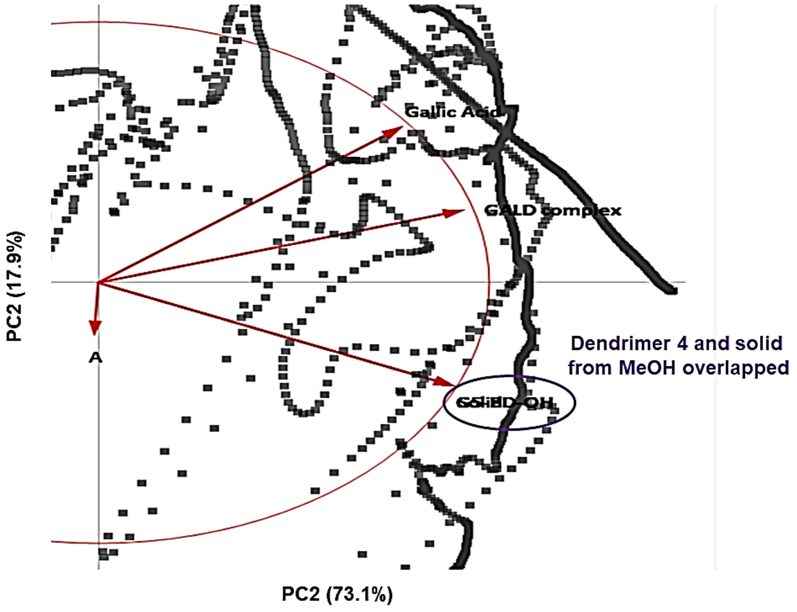

Figure S22. Bi-plot on Components PC1 and PC2 including spectral data of non-complexed molecules isolated as solid from MeOH.

Section 7. UV-Vis determination of GA concentration in GALD

Table S2. Values of A, C_{GA} and ε_{GAOxC} obtained for the six aliquots of a 31.8 μg/mL sample of GALD **7**.

A	GA (μg/mL)	ε_{GAOxC} (M^{-1} L cm^{-1})
0.2634	23.41	1913
0.2638	23.45	1913
0.2701	24.02	1912
0.2698	23.99	1912
0.2601	23.11	1914
0.2626	23.34	1913

Table S3. Data of the calibration curve: $A_{average}$ and GA standards concentrations (C_{GA}), GA predicted concentrations (C_{GAp}), residuals, absolute percentage errors and C_{GA} (μM).

C_{GA} (μg/mL)	$A_{average}$ ± SD	C_{GAp} (μg/mL)	Residuals [1] (μg/mL)	Absolute errors (%) (mg/100 mL)	C_{GA} (μM)
10	0.1039 ± 0.0138	8.9	1.1	0.11	58.8
20	0.2158 ± 0,0125	19.1	0.9	0.09	117.6
25	0.3128 ± 0.0165	27.9	2.9	0.29	147.1
40	0.4353 ± 0,0138	39.0	1.0	0.10	235.3
50	0.5522 ± 0.0122	49.5	0.5	0.05	294.1

[1] Absolute values

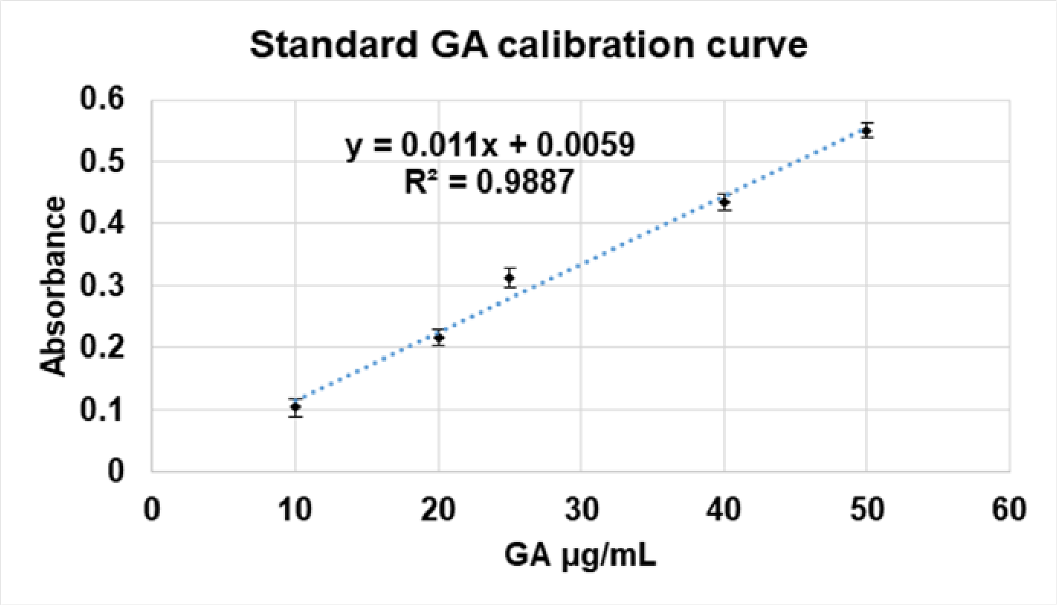

Figure S23. Standard GA calibration curve.

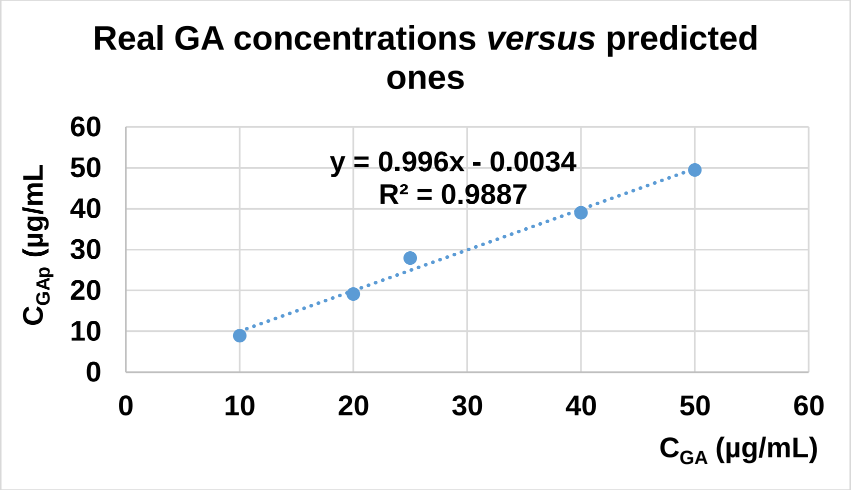

Figure S24. Real GA concentrations (C_{GA}) *versus* predicted ones (C_{GAp}).

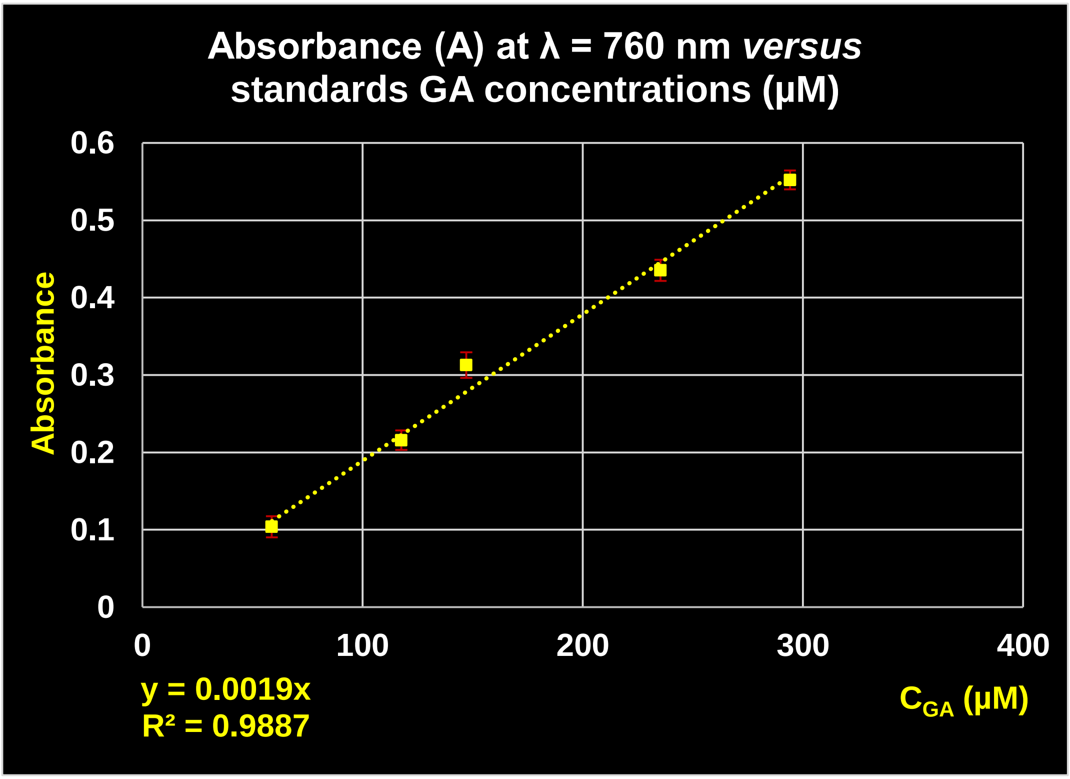

Figure S25. Absorbance (A) at λ = 760 nm *versus* standards GA concentrations (μM).

Table S4. Statistical predictive concerning calibration set, significant data of calibration, errors in the calibration and correlation coefficients.

Statistic descriptive for Calibration set [C_{GA} (μg/mL)]		Calibration	
		SEC (μg/mL); (w/v %, mg/100mL)	1.973; 0.2%
Numbers	5	RSD (μg/mL); (w/v %, mg/100mL)	0.068; 0.0068%
		SD_m (μg/mL); (w/v %, mg/100mL)	0.8823; 0.08823%
Min	10	RMSEC (μg/mL); (w/v %, mg/100mL)	1.528; 0.15%
		REC %	5.3%
Max	50	R [1]	0.9943
Media	29	R^2 [1]	0.9887
Median	25	R [2]	0.9943
Standard Deviation	15.97	R^2 [2]	0.9887

[1] Coefficient of correlation GA calibration curve; [2] Coefficient of correlation between predicted and real values.

Equations S1, S2 and S3

$$SEC\left(\frac{mg}{mL}\right) = \sqrt{\frac{\sum_{i=1}^{n}\left(C_{GA_i} - C_{GAp_i}\right)^2}{n-2}} \qquad (S1)$$

$$RMSEC\left(\frac{mg}{mL}\right) = \sqrt{\frac{\sum_{i=1}^{n}\left(c_{GA_i} - c_{GAp_i}\right)^2}{n}} \qquad (S2)$$

where C_{GAi} are the real GA concentrations, C_{GApi} are the predicted and n is the sample quantity.

$$REC\ \% = \frac{\sqrt{\frac{\sum_{i=1}^{n}\left(c_{GA_i} - c_{GAp_i}\right)^2}{n}}}{<y>}\ x\ 100 \qquad (S3)$$

where <y> is the mean value of GA concentrations of the calibration set.

Section S8. **Dynamic Light Scattering Analysis**

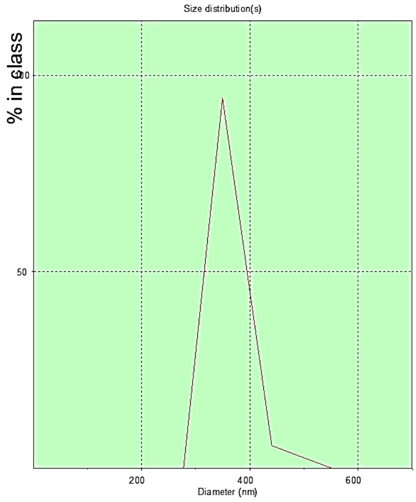

Size(nm)	Intensity	Volume	Number
55.4	0.0	0.0	0.0
69.8	0.0	0.0	0.0
87.8	0.0	0.0	0.0
110.6	0.0	0.0	0.0
139.2	0.0	0.0	0.0
175.3	0.0	0.0	0.0
220.7	0.0	0.0	0.0
277.9	0.0	14.5	18.3
349.9	94.2	39.5	43.3
440.5	5.8	35.5	31.7
554.7	0.0	10.5	6.7
698.3	0.0	0.0	0.0
879.3	0.0	0.0	0.0
1107.0	0.0	0.0	0.0
1393.8	0.0	0.0	0.0
1754.9	0.0	0.0	0.0
2209.5	0.0	0.0	0.0
2781.9	0.0	0.0	0.0
3502.5	0.0	0.0	0.0
4409.9	0.0	0.0	0.0
5552.3	0.0	0.0	0.0
6990.7	0.0	0.0	0.0
8801.6	0.0	0.0	0.0
11081.7	0.0	0.0	0.0

Peak Analysis by intensity

Peak	Area	Mean	Width
1	100.0	355.2	84.3

Peak Analysis by volume

Peak	Area	Mean	Width
1	100.0	393.2	219.5

Peak Analysis by number

Peak	Area	Mean	Width
1	100.0	379.1	198.7

Figure S26. Dynamic Light Scattering Analysis of GALD **7**: multimolecular aggregates (megamers).

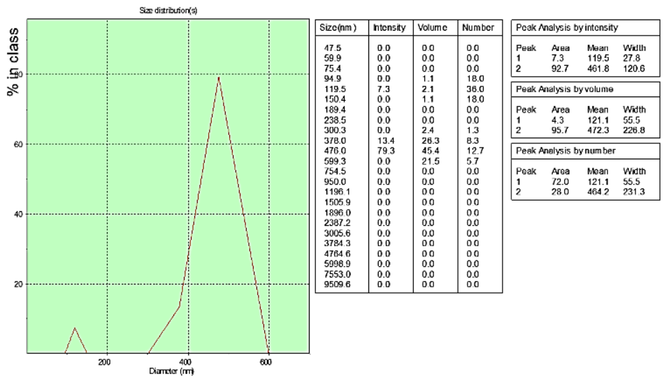

Size(nm)	Intensity	Volume	Number
47.5	0.0	0.0	0.0
59.9	0.0	0.0	0.0
75.4	0.0	0.0	0.0
94.9	0.0	1.1	18.0
119.5	7.3	2.1	36.0
150.4	0.0	1.1	18.0
189.4	0.0	0.0	0.0
238.5	0.0	0.0	0.0
300.3	0.0	2.4	1.3
378.0	13.4	26.3	8.3
476.0	79.3	45.4	12.7
599.3	0.0	21.5	5.7
754.5	0.0	0.0	0.0
950.0	0.0	0.0	0.0
1196.1	0.0	0.0	0.0
1505.9	0.0	0.0	0.0
1896.0	0.0	0.0	0.0
2387.2	0.0	0.0	0.0
3005.6	0.0	0.0	0.0
3784.3	0.0	0.0	0.0
4764.6	0.0	0.0	0.0
5998.9	0.0	0.0	0.0
7553.0	0.0	0.0	0.0
9509.6	0.0	0.0	0.0

Peak Analysis by intensity

Peak	Area	Mean	Width
1	7.3	119.5	27.8
2	92.7	461.8	120.6

Peak Analysis by volume

Peak	Area	Mean	Width
1	4.3	121.1	55.5
2	95.7	472.3	226.8

Peak Analysis by number

Peak	Area	Mean	Width
1	72.0	121.1	55.5
2	28.0	464.2	231.3

Figure S27. Dynamic Light Scattering Analysis of GALD **7**: unimolecular dendrimer particles and multimolecular aggregates (megamers).

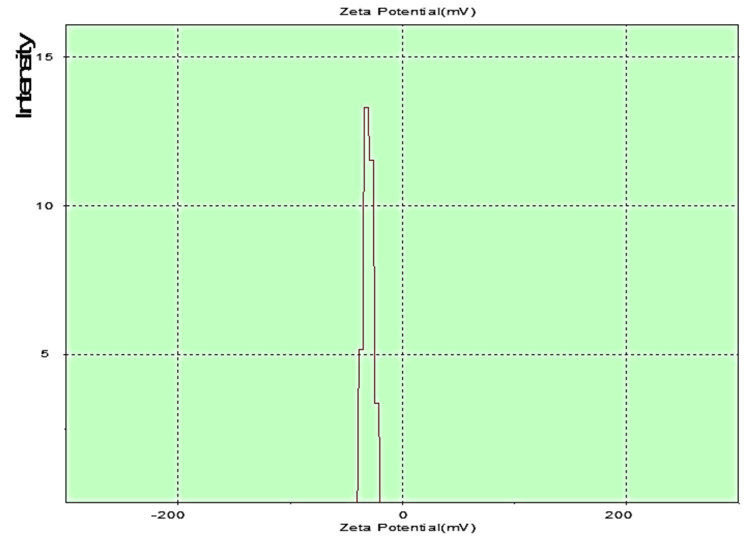

Run	Pos.	KCps	Mob.	Zeta	Width	Time
1	50.0	2071.8	-2.241	-28.5	1.6	00:19:43
2	50.0	1983.5	-2.294	-29.2	1.6	00:20:20
3	50.0	2011.9	-2.350	-29.9	1.6	00:20:56
Average		2022.4	-2.295	-29.2	1.6	
+/-		45.1	0.054	0.7	0.0	

Figure S28. Dynamic Light Scattering Analysis of GALD **7**: Z-potential.

References

[1] Alfei, S.; Castellaro, S.; Taptue, G.B. *Org. Commun.*, **2017**, *10*, 144-177.
[2] Alfei, S.; Castellaro, S. *Macromol. Res.*, **2017**, *25*, 1172–1186.
[3] Alfei, S.; Catena, S.; Turrini, F. *Drug. Deliv. Transl. Res.*, **2020**, *10*, 259-279.
[4] Alfei, S.; Oliveri, P.; Malegori, C. *ChemistrySelect,* **2019**, *4*, 8891 -8901.
[5] Alfei, S.; Signorello, M.G.; Schito, A.M.; Catena, S.; Turrini, F. *Nanoscale Adv.* **2019**, *1*, 4148-4157.
